# Tumor necrosis factor-alpha (TNF-α) enhances functional thermal and chemical responses of TRP cation channels in human synoviocytes

**DOI:** 10.1186/1744-8069-5-49

**Published:** 2009-08-20

**Authors:** Mikhail Y Kochukov, Terry A McNearney, Huaizhi Yin, Liping Zhang, Fei Ma, Larissa Ponomareva, Sarah Abshire, Karin N Westlund

**Affiliations:** 1 Department of Neuroscience and Cell Biology, University of Texas Medical Branch, Galveston, Texas USA; 2 Department of Internal Medicine, University of Texas Medical Branch, Galveston, Texas USA; 3 Department of Microbiology and Immunology, University of Texas Medical Branch, Galveston, Texas USA; 4 Department of Physiology, University of Kentucky Chandler Medical Center, Lexington, Kentucky USA

## Abstract

**Background:**

We have shown functional expression of several TRP channels on human synovial cells, proposing significance in known calcium dependent proliferative and secretory responses in joint inflammation. The present study further characterizes synoviocyte TRP expression and activation responses to thermal and osmotic stimuli after pre-treatment with proinflammatory mediator tumor necrosis factor alpha (TNF-α, EC50 1.3221 × 10^-10^g/L).

**Results:**

Fluorescent imaging of Fura-2 loaded human SW982 synoviocytes reveals immediate and delayed cytosolic calcium oscillations elicited by (1) TRPV1 agonists capsaicin and resiniferatoxin (20 – 40% of cells), (2) moderate and noxious temperature change, and (3) osmotic stress TRPV4 activation (11.5% of cells). TNF-alpha pre-treatment (1 ng/ml, 8 – 16 hr) significantly increases (doubles) capsaicin responsive cell numbers and [Ca2+]i spike frequency, as well as enhances average amplitude of temperature induced [Ca^2+^]_i _responses. With TNF-alpha pre-treatment for 8, 12, and 16 hr, activation with 36 or 45 degree bath solution induces bimodal [Ca^2+^]_i _increase (temperature controlled chamber). Initial temperature induced rapid transient spikes and subsequent slower rise reflect TRPV1 and TRPV4 channel activation, respectively. Only after prolonged TNF-alpha exposure (12 and 16 hr) is recruitment of synoviocytes observed with sensitized TRPV4 responses to hypoosmolarity (3–4 fold increase). TNF-alpha increases TRPV1 (8 hr peak) and TRPV4 (12 hr peak) immunostaining, mRNA and protein expression, with a TRPV1 shift to membrane fractions.

**Conclusion:**

TNF-α provides differentially enhanced synoviocyte TRPV1 and TRPV4 expression and [Ca^2+^]_i _response dependent on the TRP stimulus and time after exposure. Augmented relevance of TRPV1 and TRPV4 as inflammatory conditions persist would provide calcium mediated cell signaling required for pathophysiological responses of synoviocytes in inflammatory pain states.

## Background

Temperature sensitive transient receptor potential (TRP) channels belonging to the V- (or vanilloid related) subfamily are widely expressed in mammalian cells. Four members of this subfamily, TRPV1–4 conduct mono- and di-valent cations when activated by temperatures ranging from > 23°C (TRPV3 and TRPV4) to > 43°C (TRPV1) or > 53°C (TRPV2). In addition, TRPV1–4 function as important membrane sensors for extracellular chemical, osmotic, or mechanical stimuli. TRPV1 channels are activated by low pH (< 5.9) and endovanilloids. TRPV4 and TRPV2 respond to cellular swelling and mechanical stimulation. TRPV1 and TRPV4 are activated by anandamide and arachidonic acid metabolites [1-4]. Other TRP family channels also respond to chemical (icilin and camphor, TRPA1 and TRPV3) and menthol or cold stimulation (TRPM8, < 19°C). This ligand promiscuity supports an important role of TRP channels during episodes of acute or chronic inflammation, where dramatic changes in the extracellular environment impact the physiological and chemical homeostasis.

Several recent studies have demonstrated that neuronal growth factors and proinflammatory chemokines and cytokines can increase the physiologic response of TRP channels [5-8] and emphasized the enhancing role of TRP channels in chronic inflammation [3,8-10]. In a previous study [11] we showed functional expression of thermal and chemical sensitive (TRPV1, TRPV4, TRPA1) TRP channels on SW982 clonal and primary human synovial cells. We have proposed a significant role for TRP in mediation of calcium dependent proliferative and secretory responses of synoviocytes during joint inflammation. The present study supports our hypothesis demonstrating increases in thermal and osmotic sensitive TRP channel mediated responses after exposure to proinflammatory modulator tumor necrosis factor alpha (TNF-α).

TNF-α is found in abundance in synovial fluid of patients with arthritis [12], and the receptors for TNF-α are found on synoviocytes harvested from patient tissue or synovial fluid [13,14]. It is reported by Youn and colleagues [14] that upon TNF-α stimulation of synoviocytes harvested from patients, the cells proliferate and the expression of TNFR2 increases dose dependently, reaching a maximal level after 24 h of stimulation. In contrast, the levels of TNFR1 transcripts decrease up to 12 h after TNF-α stimulation in a time-dependent manner. TNF-α very effectively increases chemokine production in fibroblast-like synoviocytes harvested from arthritis patients [15]. TNF-α has been shown to be a first line initiator of inflammatory responses in joints since synovial lining cells express TNF-α prior to the appearance of other cytokines in a collagen induced arthritis model [16].

## Results

### TNF-α sensitizes synoviocyte TRPV1 to capsaicin

**Figure 1:**
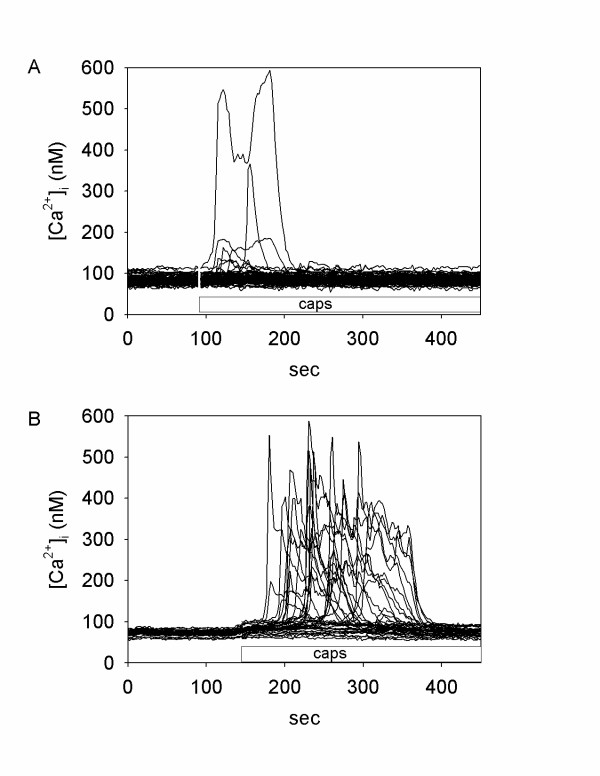
**Response of human SW982 synoviocytes to capsaicin**. Recordings of [Ca^2+^]_i _changes in human SW982 synoviocytes in response to TRPV1 agonist capsaicin (1 μM, added at the time indicated by bar). (**A**) control (vehicle treated), n = 82 cells. (**B**) TNF-α pre-treatment (1 ng/ml, 8 hr), n = 35 cells.

Synoviocyte responsiveness to a chemical TRPV1 agonist (capsaicin, 1 μM) was tested by monitoring intracellular calcium ([Ca^2+^]_i_) mobilization in control (vehicle treated) and TNF-α pretreated cells. Numerical values for the percent of cells responding, average peak amplitude and duration are provided in Additional file 1, as well as the dose response curve for TNF-α (range 10^-13 ^– 10^-6 ^g/L). Figures 1A and 1B show calcium imaging data acquired from representative control and TNF-α treated cells before and after applications of 1 μM capsaicin. Pre-treatment with TNF-α (1 ng/ml) caused a significantly increased number of individually responsive cells and increased mean spike amplitude and duration, compared to cells induced by capsaicin alone (Additional file 1). Capsaicin induced [Ca^2+^]_i _changes were completely abolished by the TRPV1 antagonist capsazepine in both control and TNF-α- treated samples, thus confirming the specificity of TNF-α-mediated vanilloid receptor sensitization. The TNF-α sensitizing effect on TRPV1 activation was evident in the concentration range of 1–50 ng/ml TNF-α after 8 to 16 hr incubations (Additional file 1). No enhancement of temperature induced responses (from > 23°C to > 43°C) occurred at lower TNF-α concentrations (10, 100 pg/ml; Additional file 1). Pre-treatment with higher doses of TNF-α (> 50 ng/ml) or longer incubation times (16 hr) did not produce further increase in the number of responsive cells or spike amplitude and duration.

### TNF-α facilitates noxious thermal-induced calcium response

**Figure 2:**
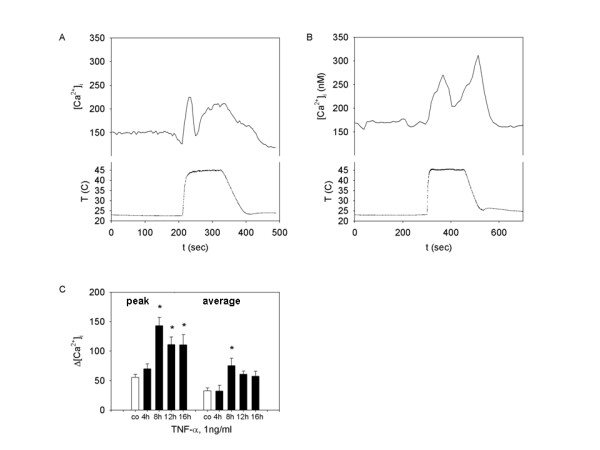
**TNF-α primed response of SW982 synoviocytes to noxious thermal challenge**. Effect of TNF-α pre-treatment on [Ca^2+^]_i _response to noxious (45°C) thermal challenge in SW982 synoviocytes. **A and B**: representative calcium traces recorded from (**A**) control (vehicle treated) synoviocytes and (**B**) TNF-α (1 ng/ml, 8 hr) pre-treated cells. (**C**) Peak and average calcium response in control (white bars, n = 7), or TNF-α (1 ng/ml) treated synoviocytes. The TNF-α was added to the incubation media for 4 hr (n = 3 trials), 8 hr (n = 4 trials), 12 hr (n = 3 trials), or 16 hr (n = 3 trials) before the experiment.* indicates a statistically significant difference (p < 0.05) assessed by ANOVA with Dunnett's test.

TNF-α induced sensitization of TRPV1 was further demonstrated by observing increased synoviocyte response to high-degree bath temperature. As shown in our earlier experiments [11], synoviocytes respond to rapid application of media heated to the noxious (45°C) range with a TRPV1 channel-mediated increase in [Ca^2+^]_i_. Figure 2A illustrates the typical synoviocyte response to prolonged 45°C hyperthermia with apparently two-waves of calcium changes. First peak and declining phases most likely represent the TRPV1 channel activation and subsequent desensitization. The nature of the second calcium wave was attributed to either gradual TRPV4 sensitization or non-specific membrane conductance changes. Therefore, we measured and reported the first [Ca^2+^]_i _peak from its initiation to the beginning of the second rising phase to quantify the TRPV1-mediated response to noxious thermal stimulation. Figures 2B and 2C, demonstrate that both maximum and average amplitudes of [Ca^2+^]_i _changes were significantly increased after TNF-α pretreatment (1 ng/ml, 8–16 hr). TRPV4 responses were characterized with hypoosmolar physiological saline (see below).

### "Moderate" temperature activates synoviocyte calcium response: Role of TRPV3 and TRPV4

**Figure 3:**
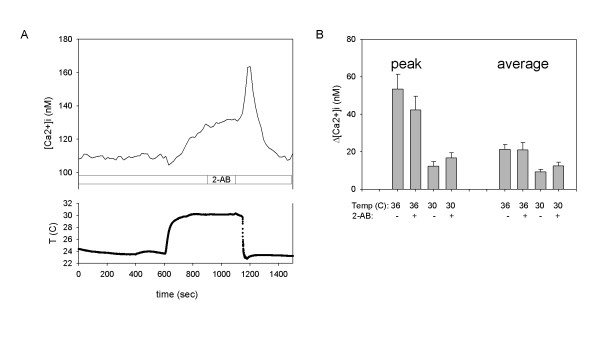
**Response of SW982 synoviocytes to "moderate" temperature increase**. Response of SW982 synoviocytes [Ca^2+^]_i _to "moderate" (30 and 36°C) temperature increase in the presence (+) or absence (-) of 2-AB. The 30 or 36°C solution was applied for 8 minutes. (**A**) Representative calcium trace shows lack of effect of 2-AB applied after 5 minutes of thermal stimulation. (**B**) In the treated groups, 2-AB was applied in the perfusion 5–10 minutes before and during thermal stimulation. Peak (left) and average (right) calcium response (n = 4 trials for each group).

Our previous data demonstrate that both TRPV3 and TRPV4 are expressed in SW982 synoviocytes, although TRPV4 mRNA expression is in relatively much greater abundance semi-quantitatively [11]. In the previous study we established the thermal threshold (28°C) to which synoviocytes respond to moderate temperature increase with increased [Ca^2+^]_i _[11]. This is characteristic for TRPV4, but concurrent TRPV3 activation at ≥ 28°C is also possible. To further characterize the specificity of synoviocyte "warm" temperature response we monitored changes in [Ca^2+^]_i _during prolonged exposure to 30 or 36°C in the presence or absence of 2-aminoethoxydiphenyl borate (2-AB) which activates and sensitizes TRPV3, but not TRPV4 [17,18]. Figure 3A, demonstrates that 2-AB applied after 5 minutes of 30°C stimulation produced no significant changes in the calcium response slope. Application of 2-AB to the bath at room temperature 5–10 minutes before thermal stimulation, did not facilitate [Ca^2+^]_i _changes caused by 30 or 35°C temperature (Fig 3B). Likewise, no change in calcium flux from baseline was noted in response to camphor (1, 3, 6, 10 mM), a TRPV3 activator, either with or without TNF-α pre-treatment (12 hr, Additional file 1). The combined studies suggest that the calcium response elicited by "moderate" (30–36°C) thermal stimulation is predominantly mediated by TRPV4, rather than TRPV3.

It is important to note that average cellular [Ca^2+^]_i _continues to rise while the temperature remains stable at 30°C. This could be explained by sensitization of thermosensitive channels during continuous stimulation. The large calcium wave occurring when the temperature is switched from 30°C to 23°C and 2-AB is removed from the chamber, could be explained by the activation of TRPM8 channels since they are reversibly inhibited by 2-AB [18]. TRPM8 responses to menthol are described below.

### TNF-α facilitates response to "moderate" temperature increase in synoviocytes

**Figure 4:**
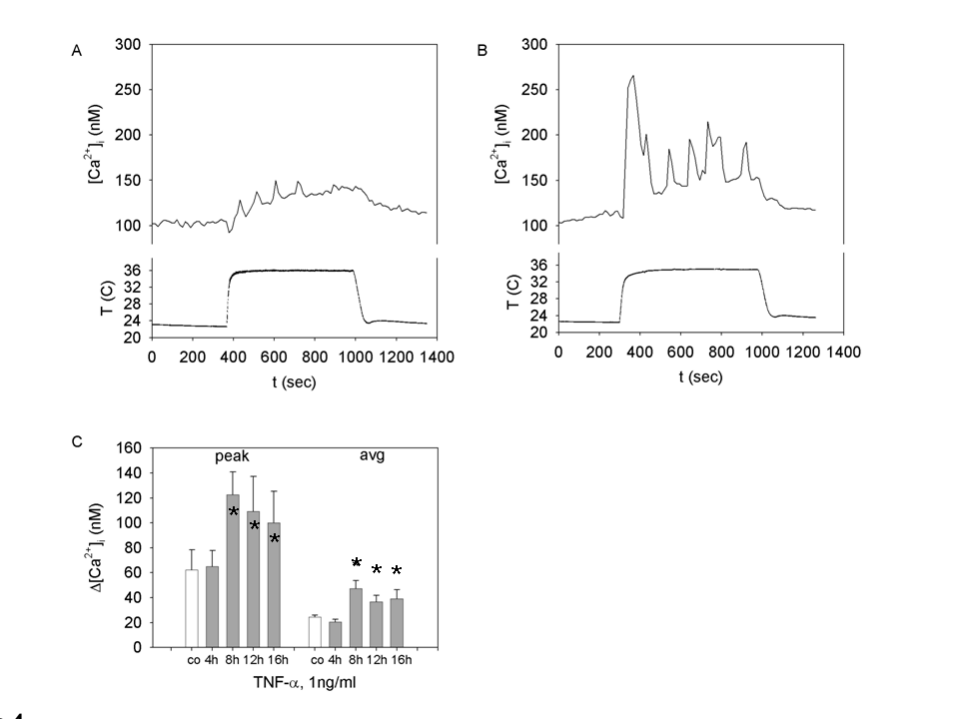
**TNF-α modulation of the SW982 synoviocyte [Ca^2+^]_i _response to 36°C**. **A and B: **Representative calcium traces recorded from (**A**) control (vehicle treated) synoviocytes and (**B**) TNF-α (1 ng/ml, 8 hr) pretreated cells. (**C**) Peak and average calcium response in control (white bars, n = 7) or TNF-α (1 ng/ml) treated synoviocytes. The TNF-α was added to the incubation media for 4 hr (n = 4 trials), 8 hr (n = 5 trials), 12 hr (n = 5 trials), or 16 hr (n = 4 trials) before the experiment. * indicates a statistically significant difference (p < 0.05) tested by ANOVA on ranks.

Pretreatment of cells with 1 ng/ml TNF-α for 8–16 hours has no effect on a temperature threshold, assessed by stepwise bath solution heating with 0.5°C per minute increments. The [Ca^2+^]_i _rise in TNF-α treated cells is observed at ≥ 28 ± 2°C,(n = 9), and is not significantly different from the temperature threshold of vehicle treated cells (29 ± 2°C) (n = 4). However, as shown in Figure 4, TNF-α caused a significant change in the magnitude of synoviocyte response to temperature stimulation above room temperature (24°C). Figure 4A shows the typical calcium response to 10 minute thermal stimulation at 36°C. Figure 4B demonstrates the typical response to stimulation at 36°C in cells also pretreated with TNF-α (1 ng/ml for 8 hr). Increased magnitude of the [Ca^2+^]_i _rise and several large peaks were observed. These calcium spikes superimposed on an elevated [Ca^2+^]_i _of significantly smaller amplitude are occasionally observed in temperature stimulated synoviocytes that were not treated with TNF-α. Both average and peak [Ca^2+^]_i _elevations induced by 36°C are larger in TNF-α treated cells, with maximal effect observed at 8 hr (Fig 4C). There was no TNF-α facilitation of the synoviocyte "cold" response.

### Specificity of temperature responses after TNF-α pretreatment

In contrast to TRPV1 thermal responses, pretreatment with TNF-α did not induce changes in synoviocyte responsiveness to "cold" activator menthol (100 μM, TRPM8) or irritant icilin (1 μM, TRPA1). Neither the numbers of cells responding to menthol (16–21%) or icilin (96–100%), nor the average amplitude and duration of the calcium response for the TNF-α-treated (1 or 50 ng/ml, 12 hr) group were significantly altered from responses of the control group (vehicle) (Additional file 1).

### Delayed osmotic stress-induced synoviocyte calcium response after TNF-α pre-treatment

**Figure 5:**
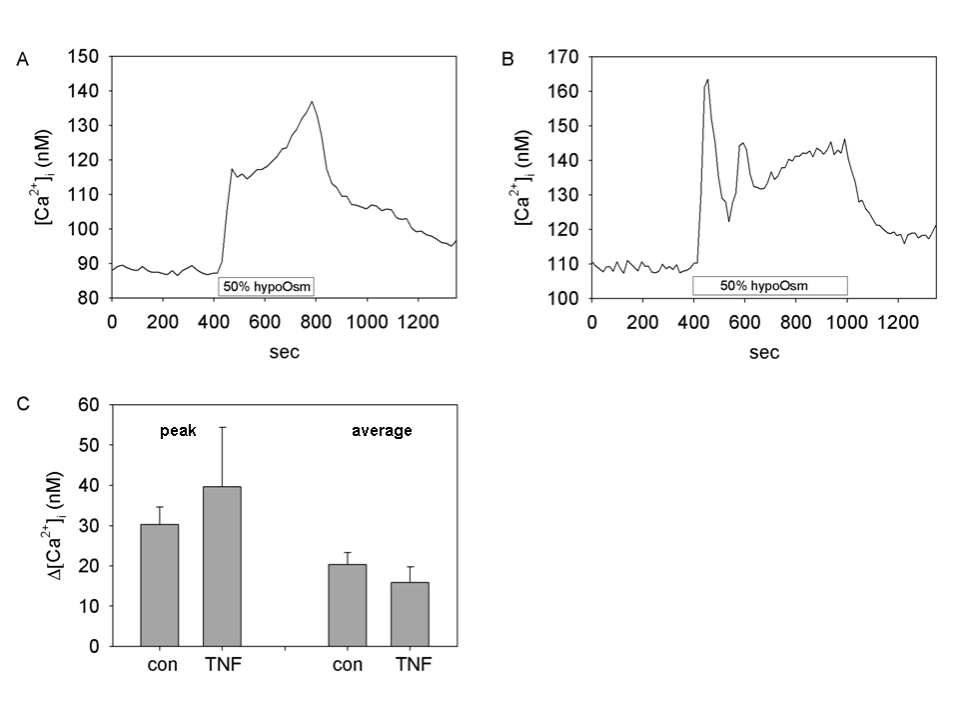
**Lack of response to 8 hr hypoosmotic challenge by SW982 synoviocytes**. Synoviocytes were poorly responsive to hypoosmotic challenge after 2, 4, or 8 hr TNF-α pre-treatment **A and B**: Representative calcium traces show responses to 50% hypoosmolar saline (150 mOsm/l) (**A**) in the absence or (**B**) presence of TRPV4 channel blocker ruthenium red (10 μM, 5 min pretreatment). (**C**) Peak and average calcium response to 50% hypoosmotic stimulation in control (n = 11 cells) or TNF-α (1 ng/ml, 8 hr) treated (n = 6 cells) synoviocytes.

It is well established that TRPV4 channels in neurons and other cells respond to decreased extracellular osmolarity and are thus considered membrane osmotic sensors. To investigate whether synoviocyte TRPV4 is sensitive to osmotic stress we initially measured calcium responses to 50% hypoosmotic challenge by rapid change of the physiological bath saline to a 150 mOsm/l solution. Figure 5A shows the typical Ca^2+ ^change elicited by hypoosmotic challenge. The small elevation of [Ca^2+^]_i _evoked is variable, is not significantly increased over average baseline response, and it is not affected by addition of the TRPV4 antagonist ruthenium red (10 μM 5 min pretreatment, Fig 5B).

Extracellular exposure to 50% hypotonic saline (150 mosmol/l) after 2, 4, or 8 hr exposure to TNF-α induces somewhat increased [Ca^2+^]_i _elevations which are variable in temporal dynamics, i.e. rapid and/or sustained as seen in control cultures. The increased [Ca^2+^]_i _plateau is observable in different cells and at different time points in the same experiment. In normal control cells and in the shorter TNF-α pretreatment experiments (2, 4, 8 hr), the average fluorescent ratio is not significantly changed by switching from isotonic (300 mosmol/l) to hypotonic (150 mosmol/l) conditions (Fig. 5C; 8 hr 0.22 ± 0.002 vs. 0.25 ± 0.017 nM, p > 0.05). Likewise, the peak responses are not elevated in TNF-α pretreated cells over responses of untreated cells exposed to hypotonic saline at shorter times (2, 4, 8 hr). Thus, in non-TNF-α-treated cells and cells pre-treated with TNF-α for 2–8 hr, TRPV4 is not responsive as a functional osmotic sensor.

**Figure 6:**
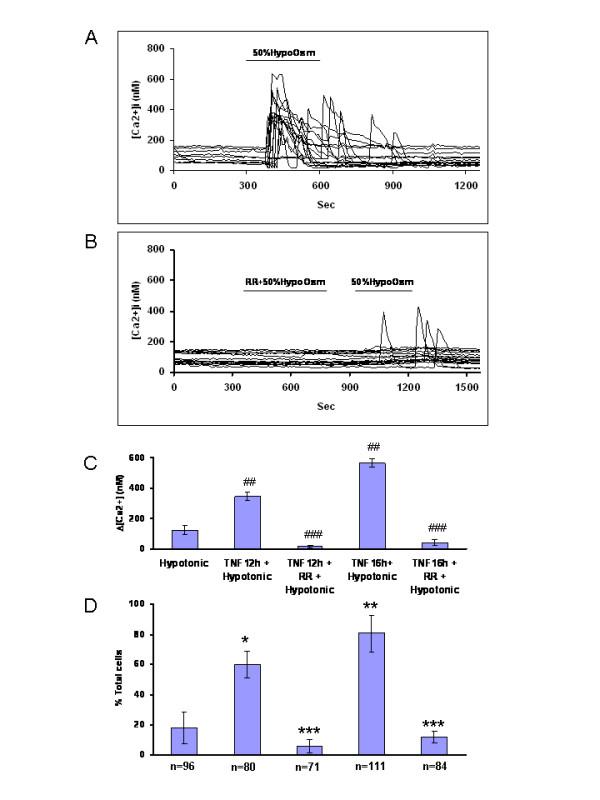
**Delayed response of SW982 synoviocytes to hypotonic challenge 12–16 hr after TNF-α pretreatment**. (**A**) Representative calcium activity traces show that after prolonged TNF-α pre-treatment (1 ng/ml, 12 hrs), SW982 synoviocytes actively responded to 50% hypotonic saline stimulation (150 mOsm/l). (**B**) A decrease in osmolarity (50%, 10 min) promotes a ruthenium red-sensitive (RR 3 μM, 10 min) intracellular calcium elevation in about 11% of the SW982 synoviocytes. TNF-α (1 ng/ml) pre-treatment for 12 or 16 hr triggers increased intracellular activity in a greater percentage of cells following hypotonic bath application (and see Additional file 2). The increased number of activated cells in hypotonic conditions is effectively blocked by bath application of ruthenium red (3 μM, 10 min). (**C**) The bar graphs show average responses (nM) of SW982 synoviocytes after different pretreatments. Hypotonic stimulation alone (50%, 10 min) does not significantly affect the average response of SW982 cells. The same hypotonic challenge in cells pre-treated with TNF-α (1 ng/ml) for 12 hr or 16 hr triggers a ruthenium red (RR)-sensitive intracellular calcium elevation. TNF-α pretreatment + hypotonic saline vs. non-treatment control + Hypotonic saline, p < 0.001 ##; TNF-α pretreatment + Hypotonic saline vs. TNF-α + RR + Hypotonic saline, p < 0.001 ###. (**D**) Pre-treatment with TNF-α significantly increased the percentage of cells responding to the 50% hypotonic challenge. The increase in responsive cells was significantly blocked by ruthenium red. TNF-α pretreatment + Hypotonic saline vs. non-treatment control + Hypotonic saline, 12 h p < 0.05 *; 16 h p < 0.005 **; TNF-α pretreatment + Hypotonic saline vs. TNF-α + RR + Hypotonic saline, 12 h and 16 h p < 0.05 ***. The X-axis marker in (**C**) and the N numbers of cells shown in (D) apply to both panels (**C**) and (**D**).

However, to investigate further whether TRPV4 is a functional osmoreceptor in conditions of inflammation, calcium microfluorometric analysis was performed in a separate study measuring changes in fluorescence emission ratio of Fura 2-loaded cells after longer exposures (12 and 16 hr) to TNF-α in clonal human SW982 synoviocytes (Fig 6 and Additional file 2). Intracellular Ca^2+ ^mobilization in response to hypoosmolarity (50%, 150 mosmol/l) was significantly elevated in cells pretreated with TNF-α (1 ng/ml) after 12 and 16 hrs compared to controls (Fig 6A, C and Additional file 2). The hypotonic triggered-calcium elevation is blocked significantly by a non-selective TRPV channel blocker, ruthenium red (RR, 3 μM) compared to the sensitized response at 12 hrs (p < 0.001) and at 16 hrs (p < 0.001) (Fig. 6B, C and Additional file 2). This indicates that TRPV4's function as an osmoreceptor is inducible in conditions of inflammation with a delayed maximal response 12–16 hr after exposure to TNF-α in these culture conditions.

### TNF-α pre-treatment (> 12 hr) increases the number of responsive synoviocytes

A larger percentage of cells respond to hypotonic stimulation after TNF-α pretreatment (Fig. 6D and Additional file 2). The percentage is significantly greater both at 12 hr (61% of total, p < 0.05) and 16 hr (81.08% of total, p < 0.005) compared to responses of the non-treated control cells (11.46% of total). The increase in number of cells reacting to hypotonic challenge can be effectively blocked by bath application of RR at the longer time points (2%, 12 hr; 13.1%, 16 hr, Fig 6D and Additional file 2). This suggests TNF-α inducible recruitment of cells with the TRPV4 osmoreceptor occurs after 12–16 hr pretreatment in the synoviocytes through downstream mechanisms.

### Specificity of TRPV4 responses after TNF-α pretreatment

While TNF-α pre-treatment (12 hr) increased responses of synoviocytes to challenges of moderate thermal and hypotonic saline, it did not promote responses to a TRPV4 agonist 4-α-phorbol 12,13-didecanoate (4α-PDD) which binds the tyrosine YS site on the third transmembrane domain to activate TRPV4. Several doses of 4α-PDD (0.1, 0.3, 1, 3 10, 30 μM, bath application) were tested [19] and no change in calcium mobilization was noted with this agent. This experiment was repeated in 6 plates, with over 130 cells selected. At the end of experiment, bath application of acetic acid (pH 5.5) onto each plate triggered a strong [Ca^2+^]_i _elevation in SW982 synoviocytes, indicating cells were viable and responsive.

### TNF-α increases expression of TRPV1 and TRPV4 mRNA, protein and immunostaining in synoviocytes after 8 hr

**Figure 7:**
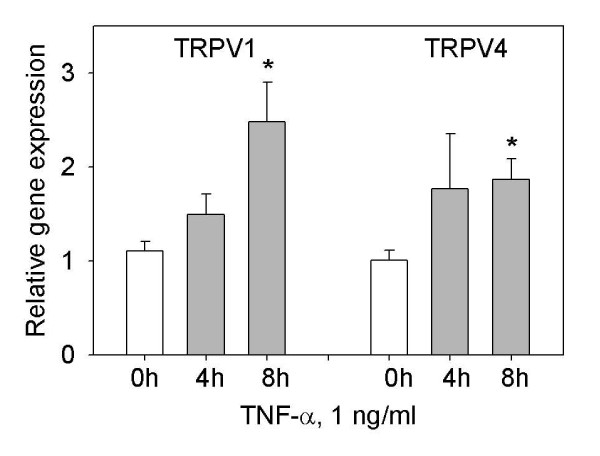
**TNF-α induces TRPV1 and TRPV4 gene expression increases in synoviocytes**. The SW982 cells when stimulated with 1 ng/ml TNF-α had increased TRPV1 and TRPV4 gene expression levels over time. The cells were harvested at 0, 4, and 8 hr for RNA extraction and analysis using quantitative RT-PCR analysis. Relative expression was compared to controls normalized at the same time point and assigned a value of 1. Each PCR reaction was normalized to the β-actin internal control. * indicates a statistically significant difference (p < 0.05) tested by ANOVA on ranks.

The increased synoviocyte sensitivity to TRPV1 and TRPV4 activators after pretreatment with TNF-α, with maximal effect at 8, 12 and 16 hr strongly suggest a TNF-α mediated up-regulation of TRPV1 and TRPV4 in SW982 synoviocytes. To assess the transcriptional effect of TNF-α treatment, quantitative real time RT-PCR experiments with TRPV1 and TRPV4 specific primers were performed. As shown in Figure 7, TNF-α (1 ng/ml) induced significant increases in TRPV1 (two-fold) and TRPV4 mRNA levels at 8 hr. The time course of the TNF-α induced TRPV1 mRNA increase is coincident with the observed increase in temperature induced calcium response.

**Figure 8:**
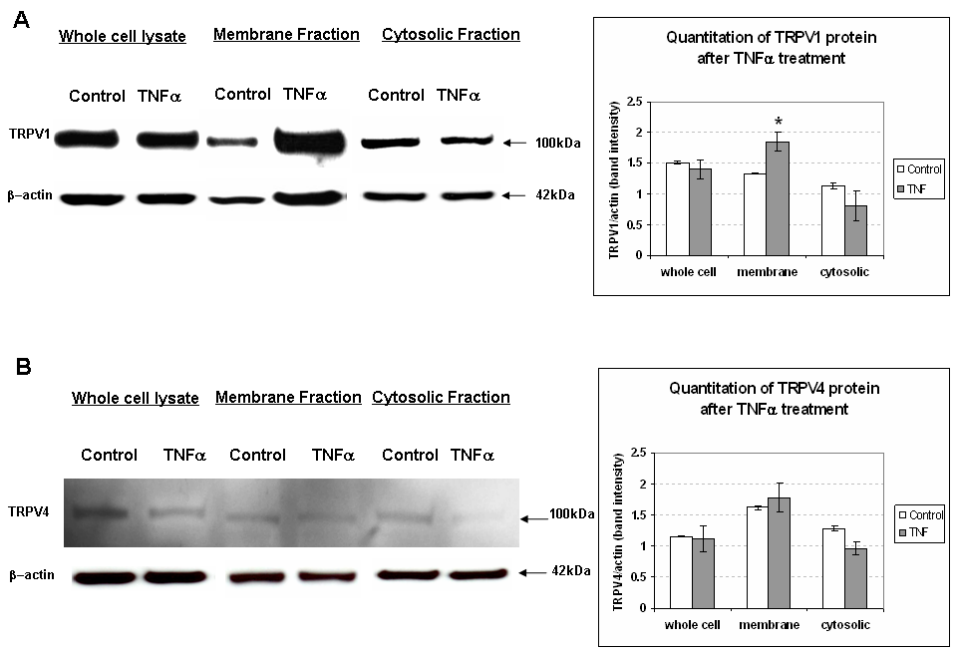
**TNF-α increases of TRPV1 protein expression in SW982 synoviocytes**. (**A**) Western Blot analysis was done on whole cell and subcellular protein fractions extracted from synoviocytes. The TNF-α stimulated increase in expression of TRPV1 protein was significant at 8 hr in the membrane fraction (n = 3 trials), implying a functional role for TRPV1 in the inflamed joint. (**B**) While TRPV4 mRNA was elevated in the RT-PCR analysis at 8 hr (**Fig. 7**), a significant increase in TRPV4 protein was not yet evident at the 8 hr time point tested. * indicates a statistically significant difference from control synoviocyte cultures (p < 0.05) tested with unpaired Student's t-test.

The mRNA increase suggested expression of TRPV1 and TRPV4 protein levels would be increased. Western blot data indicated that at the 8 hr time point, TRPV1 protein levels were elevated significantly in the membrane fraction isolated from TNF-α pretreated synoviocytes (Fig 8A), suggesting membrane translocation. TRPV4 levels were also highest in the membrane fraction but were not significantly increased at 8 hr (Fig. 8B). However, TRPV4 levels in synoviocytes were significantly increased 12 hr after pre-treatment with TNF-α compared to untreated control cells (whole cell, 0.55 + 0.008 vs. 0.44 + 0.01 a.u., p < 0.01).

**Figure 9:**
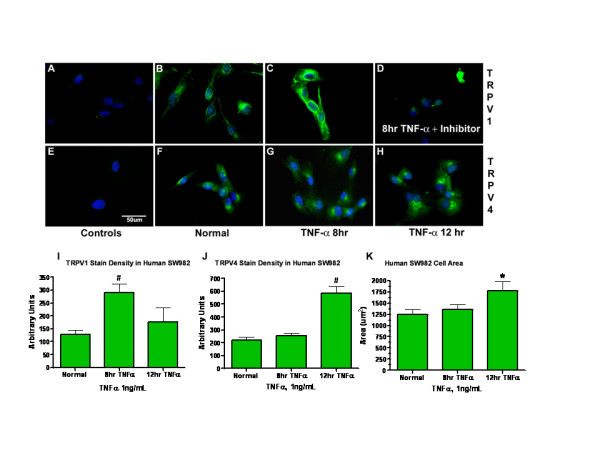
**Delayed TNF-α induced increases in TRPV1 and TRPV4**. Immunocytochemistry reveals an increase of TRPV1 and TRPV4 in human SW982 synoviocytes stimulated with 1 ng/ml TNF-α over time. (**A**) No staining is evident in peptide blocked controls: anti-TRPV1 pre-adsorbed with P-19 peptide (**B**) TRPV1 immunostaining in control cultures. An example of TRPV1 protein accumulation in a long cellular process is evident in one cell. (**C**) TRPV1 immunoreactivity is elevated after 8 hr incubations with TNF-α the TRPV1 staining has a cytoplasmic and perinuclear distribution indicating its synthesis in the rough endoplasmic reticulum. (**D**) TNF-α stimulated increase in TRPV1 staining was greatly reduced by co-incubation with TNF-α inhibitor (CAY10500, 5 μM, 8 hr). (**E**) No staining is evident in peptide blocked controls: anti-TRPV4 pre-adsorbed with peptide 853–871 (1:1, w/w, for 30 min). (**F**) TRPV4 immunostaining in control cultures. (**G**) At 8 hr, TRPV4 immunoreactivity is not increased. (**H**). TRPV4 immunostaining is enhanced after 12 hr of TNF-α stimulation compared to untreated cell cultures. (**I**) TRPV1 stain density was significantly increased at 8 hr. Other time points were observed visually, but not quantified. (**J**) TRPV4 stain density was significantly increased at 12 hr and significantly decreased at 16 hr. (**K**) The bar graph indicates that the synoviocyte cell areas are significantly increased at the same time point when TRPV4 staining is increased (12 hr). * indicates p < 0.05 compared to normal synoviocytes. # indicates p < 0.01 compared to normal synoviocytes. Data is representative of n = 3 experiments.

Immunocytochemical localization of TRPV1 and TRPV4 was examined after 8 and 12 hr incubations with TNF-α (Fig 9). TRPV1 immunostaining in SW982 synoviocytes was significantly elevated only at the 8 hr incubation time point after TNF-α compared to TRPV1 in normal cells (289 ± 34.8 vs. 127 ± 4.9 arbitrary units, a.u., p < 0.01) (Fig 9C, I). TNF-α stimulated increase in TRPV1 staining was greatly reduced by co-incubation with TNF-α inhibitor CAY-10500 (5 μM, 8 hr) (Fig. 9D). Increase in TRPV4 immunostaining was not significantly elevated at 8 hr (252 ± 20.0 a.u.) (Fig 9G, J), but was increased at the 12 hr time point (584 ± 53.7 vs. 221 ± 21.6 a.u., p < 0.05) (Fig 9H, J). Cytotoxicity of the CAY-10500 inhibitor with the prolonged 12 h incubations did not allow testing for reduction of the TRPV4 increase seen at 12 hr. The TRPV1 and TRPV4 were localized around the nucleus in rough endoplasmic reticulum indicating synthesis, as well as in the cell cytoplasm. Membrane localization is supported by the functional [Ca^2+^]_i _and Western blot cell fraction data. After 12 hr TNF-α treatments, SW982 synoviocytes assumed a more flattened appearance with an increase in cell area (1772 ± 203.1 vs. 1246 ± 106.1 um^2^) (Fig 9K) and observed nuclear area, suggesting increased cellular activity that corresponds with the perinuclear staining and increased responsiveness. Average cell diameter was significantly increased at 12 hr (Fig. 9I–K) and cells resume their original size and stain density 16 hr after TNF-α treatment. Cell and nuclear integrity are maintained throughout all time points, and cell proliferation continues.

The SW982 synoviocyte staining for TRPV1 is coincident with the peak physiological responses (8 hr). The delay in increased responsivity to TRPV4 (Fig 6) occurs at the peak of expression, staining and cell area increases (12 hr).

## Discussion

In the present study we demonstrated up-regulation of two members of the TRP cation channel family in SW982 human synovial cells after preincubation with TNF-α. The TNF-α induced increases in TRPV1 and TRPV4 gene and protein expression at 8 and 12 hr respectively, are coincident with significant enhancement of responses to increasing temperature and decreasing osmolarity mediated by TRPV1 and TRPV4. These two TRP channels appear to be the major thermal and osmosensors in synoviocytes. Marginal facilitation of the calcium response at 30°C, in synoviocytes incubated with 2-AB demonstrates the potential for TRPV3 mediated response to thermal stimuli in some circumstances, although no response to camphor was generated. TNF-α did not induce a change in synoviocyte responsiveness to TRPA1 (icilin) or TRPM8 (menthol) activation.

A number of endogenous factors can activate TRPV1 and TRPV4 during inflammation (for review see [3,10]). This includes factors generated in vast amounts during inflammation, i.e. direct ligands such as protons, temperature increases, lipid mediators and hypoosmolarity, or indirect activators such as cytokines, bradykinin, ATP, serotonin or PGE2 [8]. We have previously shown responsiveness of synoviocytes to capsaicin, resiniferatoxin and icilin [11]. The increased levels of extra-physiologically activated TRPV1 and TRPV4 channels caused by incubation with TNF-α can significantly contribute to the activation of synoviocytes observed in joint inflammation. Like other fibroblasts, the fibroblast-like synoviocytes secrete TNF-α [20], as well as express its receptors [13]. It is highly likely that TNF-α increases expression of TRPV1 and TRPV4 by mediating the up-regulation directly and/or through initiation of downstream events since the inhibitor reduced the immunostaining increase. Further studies are required, however.

Sensitization of TRPV1 and TRPV4 was demonstrated after TNF-α exposures with increased cell responsiveness to chemical agonist capsaicin, noxious thermal and hypoosmolar stimulation. It is still obscure, why only a fraction of the cultured clonal synoviocytes had increased responsiveness to capsaicin, as all cells demonstrated increased [Ca^2+^]_i _in response to noxious temperature and icilin. It is also unclear why the synoviocytes readily responded to hypoosmolarity but not to TRPV4 agonist 4α PDD. This heterogeneous response suggests that these TRP channels exist in multi-potential functional states, that non-neural cellular responses are not identical to neuronal responses, and/or that tertiary protein structure plays a role in activation properties. Other mechanisms regulating TRP channel sensitization may be involved, such as PKC or PKA dependent phosphorylation [8,9,21-24] or phosphatidylinositol phosphate binding [1,25]. Further studies detailing the molecular mechanisms of TNF-α induced increase of TRPV1 and TRPV4 mediated response in synoviocytes are needed.

Specificity of the TNF-α induced sensitization for TRPV1 and TRPV4 responses was demonstrated. No significant change in calcium flux from baseline and numbers of synoviocytes responding was noted after TNF-α pre-treatment (12 hr) for menthol, a TRPM8 activator or camphor, a TRPV3 activator. While almost all of the cells responded to icilin, TNF-α pre-treatment did not alter the response properties of the synoviocytes to the TRPA1 stimulation. Lack of response to camphor and lack of sensitization of the menthol response provide evidence in agreement with Kochukov et al. [11] that TRPV3 and TRPM8 are minimally expressed in this human SW982 synoviocytes cell line. Thus, TNF-α sensitized thermal and hypoosmotic responses of the synoviocytes are mediated primarily by TRPV1 and TRPV4 activation respectively.

The present study was performed on the human SW982 synovial sarcoma cell line, which a number of studies have shown share similar physiological properties, cytokine expression and regulation profiles with primary human type B synovial cells. Our previous data demonstrate the functional expression of TRPV1, TRPV4 and other TRP channels in both SW982 cells and primary human synoviocytes [11]. In all aspects the results were similar for the clonal and primary cells. The presence of functional TRPV1 in human synovial and dermal fibroblasts has been confirmed by other authors [26,27]. In a separate study we also demonstrated close similarity between primary human synoviocytes and SW982 cells in the functional expression of a G-protein coupled membrane acid sensing receptor [28].

Many previous studies describing TRP channel up-regulation have been performed on neuronal cells, where TRPV1 and/or TRPV4 sensitization are shown to be an important mechanism contributing to chronic hyperalgesia [8,29-33], bronchial hyper-responsiveness [34], neurogenic bladder [35], and neurogenic inflammation [9,36-39] (for detailed review [3,10]). In contrast, TRP channel function and regulation in non-neuronal cells such as synovial fibroblasts are less well studied, although the physiologic contribution of TRPV1 in experimental arthritis has been well documented recently using TRPV1-knockout mice [8,31,40-42]. Abundant expression of TRPV1 in human synovial cells and its up-regulation in the membrane fraction with prolonged TNF-α exposure support our hypothesis that non-neuronal TRP channels are participants in development of peripheral inflammation.

Our current study demonstrates increased TRPV4 mediated calcium mobilization in response to hypotonicity in synoviocytes. However, we did not initially observe significant changes in hypotonicity-induced calcium response in synoviocytes after TNF-α co-stimulation at 2, 4, and 8 hr, despite the moderate temperature sensitization. Upon further study, however, the functional consequences of TRPV4 up-regulation by TNF-α become evident after 12 hr and persisted through 16 hr of study. The average amplitude of the TRPV4 activated calcium responses was significantly elevated and was blocked by ruthenium red. The numbers of cells responding to hypoosmotic stimuli were greatly increased 3-fold at 12 hr and 4.5-fold at 16 hr.

The role of TRPV4 in synoviocytes is less clear as is the delay in activation response. Edema is a cardinal sign of inflammation and osmolarity changes may be a factor. The delayed TRPV4 sensitization at 12 hr was coincident with increased TRPV4 immunostaining and protein content, and development of a flattened appearance of the cells. These increases became evident when TRPV1 staining had returned to normal levels. A universal role for TRPV4 in osmosensation and mechanotransduction has been promoted in recent studies based on emerging data from neural cells, epithelia, mesenchymal cells, kidney and osteoclasts ([2], reviewed in [43]). It is noted in neurons that simultaneous action of a number of combined inflammatory mediators is required to achieve sufficient activation of the cAMP pathway to allow TRP-dependent hyperalgesia to occur [8]. The present study demonstrates that the inflammatory mediator TNF-α which releases a cascade of other mediators and enzymes is sufficient over time to promote the shift in synoviocytes to osmoreceptor mediated through TRPV4.

Surprisingly, the TRPV4 response to 4α PDD was absent in the human synoviocytes. This difference may be explained by distinctive properties of synoviocyte TRPV4 as heterogeneity of this channel due to alternative splicing and heteromultimerization has been reported [44]. The TRPV4 transmembrane segment TM3 is a crucial region for the activation by its most specific agonist 4 alpha-phorbol 12, 13-didecanoate (4α-PDD). Vriens et al. [45] have found that mutation of one tyrosine (Tyr555) at the intracellular N terminal of TM3 (S3) leads to a loss of responsiveness to 4α-PDD, despite the demonstrated presence of a functional channel activated by hypotonic stimulation. This suggests inability of 4α-PDD binding at the single critical tyrosine in the TM3 segment in human synoviocytes likely due to the tertiary folding of the protein in this region as suggested previously [45].

The present data indicate that synoviocytes express functional thermal and osmotic receptors inducible under inflammatory conditions. The TNF-α mediates up-regulation of TRPV1 and TRPV4, doubling mRNA and protein expression at 8 and 12 hr, respectively. Microarray data has been published detailing downstream targets for TNF-α in synoviocytes [46] and demonstrates regulation of numerous genes by multiple pathways. Increased expression of vanilloid channels in the membrane fraction in the present study implies translocalization of TRPV1 and TRPV4 to the plasma membrane of synoviocytes initiated in response to the TNF-α. The time course of TNF-α induced increase in TRPV1 and TRPV4 protein expression correlates well with the observed TNF-α induced increases in thermal and hypoosmotic induced [Ca^2+^]_i _responses and cell recruitment.

The literature suggests that TRP expression in epithelial lining cells may be extremely important in cells that interface with dramatic changes in the external and internal environment induced by thermal and mechanical stress, acidity, hypoosmolarity, foreign chemicals and endogenous mediators. Synoviocytes are exposed to all of the above stimuli in the course of routine joint function and in acute and chronic inflammatory states. The synoviocytes are like other fibroblasts that secrete the inflammatory mediator, TNF-α [20], as well as express TNF-α receptors [13,14]. It is known that the TNF-α can directly activate TNF-α receptors on peripheral nerve terminals to amplify hyperalgesic responses [47,48] and release enzymes causing injurious joint destruction and impaired biomechanical function [49-51]. The TRPV1 and TRPV4 mediated [Ca^2+^]_i _signaling responses of synoviocytes during inflammation would participate in the TNF-α mediated functions that promote the painful inflammatory and joint destruction processes locally and impact subsequent responses of the immune system. The data suggest TRPV1 and TRPV4 play important roles mediating the intracellular Ca^2+ ^increase that drives critical functions such as associated proliferative and secretory responses of synoviocytes during joint inflammation thus promoting TRPV1 and TRPV4 as attractive potential therapeutic targets.

## Conclusion

This paper confirms a physiological role for TRP cation channels in transduction of relevant information to synovial lining cells. The inflammatory milieu includes thermal, osmolar, acidic and mediator stimuli, all potent activators of TRP channels. Our study supports an inducible responsivity for TRP cation channels TRPV1 and TRPV4 in synoviocytes to inflammatory mediator, TNF-α. Pretreatment of human synoviocytes with TNF-α produces (1) increased TRPV1 and TRPV4 mRNA expression at 8 hr, (2) increased TRPV1 protein expression at 8 hr in the membrane fraction, (3) increased TRPV4 cellular expression and increased cell size at 12 hr, and (4) differentially enhanced calcium responsiveness and cell recruitment in response to thermal and osmolarity challenges. Responses of synoviocytes depend on the TRP stimuli and time point after TNF-α exposure. The delayed transduction and responsivity are highly suggestive of downstream transcriptional regulation. This study emphasizes the need for studies designed with prolonged time courses to better understand clinically relevant physiological responses of local cells that impact inflammatory pain states. These findings further support augmented relevance of synoviocyte TRP ion channels in arthropathies, and potentially other non-neuronal TRP responses in inflammatory states.

## Methods

### Cell lines and cultures

The fibroblast-like synovial SW982 [42] cells were obtained from the American Type Culture Collection (ATCC, Bethesda, MD) and maintained in Leibovitz's L-15 medium (GIBCO, Grand Island, NY), 10% heated fetal bovine serum (FBS, Gemini Bio-Products, Woodland, CA), 2 mM L-glutamine, 1,000 U/ml penicillin G (GIBCO), and 100 ng/ml streptomycin (GIBCO). Cells were incubated in a humidified cell incubator (37°C, ambient CO_2_). Low passage cells (3–15×) were used in this study.

### Cytosolic free calcium ([Ca^2+^]_i_) measurement

For measurement of [Ca^2+^]_i _in cells exposed to chemical activators of different TRP channels a fluorescent calcium imaging approach was used. Cells were harvested with 0.25% trypsin-0.02% EDTA disruption, plated on 15-mm circular quartz glass coverslips at a density of 200,000 cells/mm^3 ^and incubated at 37°C for 24–48 h before an experiment. On the day of the experiment, the cells were loaded with the calcium-sensitive fluorescent dye fura-2 as previously described [11]. Fluorescent recording of temperature-induced calcium changes was performed as described earlier [11] using Nikon Diaphot microscope with an 60× water immersion lens and Till Photonics Polychrome II photometry setup (Munich, Germany) equipped with Hamamatsu R928 photomultiplier and CL-100 bipolar temperature controller with a SC-20 inline solution heater/cooler (Warner Instruments, Hamden, CT) and controlled by ITC-18 computer interface (Instrutech, Port Washington, NY) and X-chart software(HEKA, Heidelberg, Germany). Experiments were repeated a minimum of four times each. The 340-to-380 ratios acquired from single or two-three neighboring cells every 200 ms were converted into [Ca^2+^]_i _with the formula [Ca^2+^]_i _= *K*_d _[(R-R_min_)/(R_max _- R)](S_f2_/S_b2_)[52], in which fluorescence ratio sat zero free Ca^2+ ^(R_min_) and saturation free Ca^2+ ^(R_max_), as well as fluorescent intensity of Ca^2+^-free (S_f2_) and Ca^2+^-bound(S_b2_) dye, excited at 380 nm, were measured experimentally and fura-2 *K*_d _values for Ca^2+ ^at different temperatures were calculated in accordance Shuttleworth and Thompson estimations [53]. Each calcium trace was evaluated individually and the response was considered positive when the elevation in [Ca^2+^]_i _was clearly seen at the appropriate time and when the [Ca^2+^]_i _returned to baseline in the absence of treatment. Detection threshold of [Ca^2+^]_i _changes was dictated by signal to noise ratio and was at least 10 nM over the baseline. Peak and average amplitude of [Ca^2+^]_i _changes over a baseline were used for quantitative estimation of calcium response.

For simultaneous measurement of [Ca^2+^]_i _in large numbers of cells exposed to chemical activators of different TRP channels, human SW982 synoviocytes were plated onto 12 mm coverslips, cultured for 24 hr, then pre-treated with TNF-α (1 ng/ml). The cells were loaded with the calcium-sensitive fluorescent dye fura-2 for 30–60 min before calcium recording. The activation was done on 3 plates with over 60 cells selected. The imaging setup included a Nikon 200E microscope with a 20× SuperFluo lens, and a computer-controlled illumination system (Sutter Instruments, Novato, CA) equipped with a digital monochrome-cooled charge-coupled device RoperCoolsnap HQ camera (Roper Scientific, Tucson, AZ). Fluorescent emission at 510 nm from regions of interest (corresponding to a single cell) was acquired online with the MetaFluor software (Universal Imaging, Downington, PA). The signal was obtained in dual, 340 and 380 nm, excitation mode and average intensity of fluorescence in each region after were used to estimate 340-to-380 ratios. The elevation in calcium influx for the individual and population data was considered an increased response if statistically different from the baseline.

### Chemicals

The fluorescent intracellular calcium release indicator fura-2AM was obtained from Molecular Probes (Eugene, OR). TRPV1 agonist (capsaicin) and antagonists (capsazepine and 2-aminoethoxydiphenyl borate (2-AB; also antagonizes TRPV3 but not TRPV4, inhibits TRPM8), were obtained from Tocris (Ellisville, MO), as was the icilin (TRPA1 agonist). The ruthenium red (RR, TRPV1 and TRPV4 antagonist), 4-α-phorbol 12, 13-didecanoate (4α-PDD, TRPV4 antagonist), and camphor (TRPV3 agonist) were obtained from Sigma (St. Louis, MO). L-menthol (TRPM8 agonist) was obtainedfrom Aldrich (Milwaukee, WI). The inflammatory cytokine tumor necrosis factor alpha (TNF-α) was obtained from Calbiochem (San Diego, CA). The TNF-α inhibitor CAY10500 (6,7-dimethyl-3-{[methyl-[1-(3-trifluoromethyl-phenyl)-1 H-indol-3-ylmethyl]-amino}-ethyl)-amino]-methylchromen-4-one) was obtained from Cayman Biologicals (Ann Arbor, MI). Thapsigargin used to test cytosolic calcium release at the end of each study was obtained from Calbiochem (La Jolla, CA).

### Real time PCR

The low passage SW982 cell cultures were grown in 75 mm flasks until they reached 70–80% confluency. Leibovitz's L-15 Medium (Invitrogen, Carlsbad, CA) with 10% FBS, 1% P/S was used for culture medium and the cells were incubated at 37°C, room air, per ATCC recommendations. For the experiments, TNF-α (1 ng/ml) was added to the designated flasks. The cells were harvested by disruption (see below) over a time course of baseline (immediately after TNF-α addition), and at 4 and 8 hours after addition. Flasks containing untreated cells were also harvested at the same time as baseline controls. These studies were performed in three separate experiments.

Total RNA was isolated using Trizol reagent (Cat# 10296028, Invitrogen) in accordance with manufacturer's protocol. First-strand cDNAs were synthesized from total RNA by RT using the SuperScript^® ^III First-Strand kit (Cat# 18080051, Invitrogen) with random hexamer primers as described by the manufacturer. Two microgram of total RNA from each individual sample was used to synthesize cDNA for TRPV1, TRPV4 and β-actin, respectively.

Quantitative real-time PCR analysis was performed by the use of an Applied Biosystems 7000 Sequence Detection System (Applied Biosystem Inc, Foster City, CA). Each sample was analyzed in triplicate and the means were used for statistical purposes. The thermal profiles were obtained by using 2 min incubation at 50°C, followed by an initial 10 min denaturation step at 95°C, and by 40 cycles of 1 min each at 60°C plus 15 sec at 95°C. Significant contamination with genomic DNA was excluded by amplifying non-reverse-transcribed RNA.

The probes and primers for TRPV1 (Hs00218912_m1), TRPV4 (Hs00540967_m1) and β-Actin (Hs99999903-m1) were ordered from Applied Biosystem Inc. The threshold cycle (Ct), i.e. the cycle number at which the amount of amplified gene of interest reached a fixed threshold, was determined subsequently. Relative TRPV1 and TRPV4 mRNA expression was calculated by the comparative Ct method described elsewhere ()[54]. Analysis of relative gene expression data using real time quantitative PCR and the 2-Ct method. The relative quantitative value of target, normalized to an endogenous control β-actin gene and relative to a control, is expressed as 2^-ΔΔCt ^(fold), where ΔCt = Ct of target gene (TRPV1 or TRPV4)-Ct of endogenous control gene (β-actin), and ΔΔCt = ΔCt of samples for target gene-ΔCt of the control for the target gene. 2 μl of synthesized cDNA from each individual sample was used to amplify for TRPV1, TRPV4 and β-actin, respectively.

### Western blot analysis

Low passage SW982 cell cultures were grown in 75 mm flasks until they reached 70–80% confluency. Proteins were extracted from control cultures without treatment and after treatment with 1 ng/ml TNF-α (Pierce Biotechnology, Inc., Rockford, IL) for 8 hours. Western Blot analysis was performed on whole cell protein and subcellular protein fractions extracted from synovial SW982 cells with ProteoExtract Subcellular Proteome Extraction kit (Calbiochem). Protein transferred to membranes were probed with anti-TRPV1 antibody (VR1, P-19, 1:1000, Santa Cruz, Burlingame, CA) and TRPV4 (VR4, K-18, 1:200, Santa Cruz) antibody, at 4°C overnight. Amounts of total protein loaded per lane: 15 μg for cytosolic and membrane fractions, 10–15 μg for whole cell and 5 μg for rat spinal cord lysate as a positive control. The protein analysis was performed with protein from three experiments.

### Immunocytochemistry

Cells on glass coverslips were fixed with 4% freshly mixed paraformaldehyde:methanol (5:1, v/v) for 30 min at room temperature. The coverslips were washed 3× in PBS and incubated at room temperature for 30 minutes with 3% neutral serum. The primary antibody (anti-TRPV1, (1:500, P19: sc-12498, Santa Cruz, Burlingame, CA) or TRPV4 (1:400, #ACC-034, Alomone, Jerusalem, Israel) was diluted in TBS with 1.0% neutral serum (0.02% Triton X-100). The primary antibody was allowed to incubate on the glass coverslips for 48–72 h at 4°C. The glass coverslips were then washed 3× with 1% serum, 0.02% Triton X-100 and then incubated for 30–60 min at room temperature with the appropriate secondary IgG antibody (1:200, 1 hr), using donkey anti-goat (for TRPV1) and goat anti-rabbit (for TRPV4). Each had a FITC fluorescent tag (1:200, Santa Cruz). After final washes 3× with PBS, the stained coverslips were inverted onto a dot of VectaShield mounting media containing blue nuclear counterstain DAPI. Immunocytochemical controls included the absence of the primary or secondary antibodies, or were done in the presence of matched serum at the same dilution. The specificity of the antibodies was confirmed for TRPV1 by adsorption controls with P-19 N-terminus peptide provided by Santa Cruz, and for TRPV4 with peptide 853–871 supplied by Alomone Labs (1:1 w/w, 30 min). There was no staining in either the method controls or the peptide blocked controls. TNF-α stimulated increase in TRPV1 was greatly reduced by co-incubation with a TNF-α inhibitor, CAY10500. Stained cells were visualized with a Nikon FXA microscope equipped with MetaVue software (Nikon Instruments, Inc. Melville, NY).

### Software and statistics

Sigma Plot and Sigma Stat scientific software (SPSS, Chicago, IL) were used for conversion and analysis of acquired data. Data are reported as means ± SE. MetaMorph software (Universal Imaging, Downington, PA) were used for off-line analysis of calcium imaging data. Student's t-tests or ANOVA for multiple comparisons with Tukey's multiple comparison post hoc test or Dunnett's method were used to compare data obtained from TNF-α treated vs. control series, unless otherwise indicated.

## Competing interests

The authors declare that they have no competing interests.

## Authors' contributions

All authors read and approved the manuscript. MYK was responsible for calcium imaging responses to capsaicin, thermal and osmotic challenges, as well as the initial manuscript draft; TAM was responsible for experimental and primer design for synoviocyte culture studies; HY was responsible for RT-PCR; LZ and FM were responsible for all calcium imaging studies with TNF-α primed synoviocytes; SA and LP were responsible for western blots of whole cell and cell fractions of control and TNF-α primed synoviocytes; SA was responsible for immunolocalization studies, microscopy, cell area and MetaMorph analyses; and KNW was responsible for experimental design of the studies, data synthesis, data figures and manuscript editing for publication.

## Supplementary Material

Additional file 1 **Responses of human SW982 synoviocytes to capsaicin, icilin and menthol after TNFα pre-treatment. **Dose response curve for TNF-α pre-treatment (8 hr) and stimulation with capsaicin (1 μM). Click here for file

Additional file 2 SW982 synoviocytes responses to hypotonic saline, with and without pre-treatment with TNF-α, with and without inhibitor ruthenium red (3 μM). Click here for file
