# Supplementary material for: Tumor necrosis factor-alpha (TNF-α) enhances functional thermal and chemical responses of TRP cation channels in human synoviocytes
Source: Mol Pain. 2009 Aug 20;5:49. doi: 10.1186/1744-8069-5-49 (PMC3152771; doi:10.1186/1744-8069-5-49)
Supplement: Additional file 1 — Responses of human SW982 synoviocytes to capsaicin, icilin and menthol after TNFα pre-treatment. Dose response curve for TNF-α pre-treatment (8 hr) and stimulation with capsaicin (1 μM). [file 1744-8069-5-49-S1.doc]

|  | **% cells responding**  **(total number of cells tested)** | **Average amplitude (peak ΔCa ± s.e.m. nM)** | **Average duration of Ca peaks (sec)** |
| --- | --- | --- | --- |
| **Control** | **40 (82)** | **47 ± 16** | **79 ± 4** |
| **TNF-α 10 pg/ml 8 hr + capsaicin** | **46 (41)** | **44 ± 19** | **86 ± 8** |
| **TNF-α 100 pg/ml 8 hr**  **+ capsaicin** | **53 (43)** | **81 ± 22** | **101 ± 16** |
| **TNF-α 1 ng/ml 8 hr + capsaicin** | **89 (35)** | **248 ± 24*** | **105 ± 11** |
| **TNF-α 1 ng/ml 16 hr + capsaicin** | **80 (46)** | **194 ±30*** | **110 ± 9*** |
| **TNF-α 50 ng/ml 8 hr**  **+ capsaicin** | **87 (52)** | **239 ± 18*** | **110 ± 6*** |

| **Control** | **96 (70)** | **68 ± 14** | **85 ± 6** |
| --- | --- | --- | --- |
| **TNF-α, 1 ng/ml 12 hr**  **+ icilin** | **95 (34)** | **51 ±21** | **81 ± 12** |
| **TNF-α, 50 ng/ml 12 hr + icilin** | **100 (38)** | **57 ± 22** | **77 ± 12** |

| **Control** | **16 (37)** | **33 ± 10** | **79 ± 6** |
| --- | --- | --- | --- |
| **TNF-α, 1 ng/ml 12 hr**  **+ menthol** | **15 (27)** | **39 ±16** | **77 ± 11** |
| **TNF-α, 50 ng/ml 12 hr + menthol** | **21 (33)** | **31 ± 12** | **70 ± 8** |

*** p<0.05**
