# Supplementary material for: Tumor necrosis factor-alpha (TNF-α) enhances functional thermal and chemical responses of TRP cation channels in human synoviocytes
Source: Mol Pain. 2009 Aug 20;5:49. doi: 10.1186/1744-8069-5-49 (PMC3152771; doi:10.1186/1744-8069-5-49)
Supplement: Additional file 2 — SW982 synoviocytes responses to hypotonic saline, with and without pre-treatment with TNF-α, with and without inhibitor ruthenium red (3 μM). [file 1744-8069-5-49-S2.doc]

|  | **% cells responding**  **(total number of cells tested)** | **Average amplitude**  **(peak ∆Ca ± s.e.m. nM)** | **Average duration of Ca peaks (sec)** |
| --- | --- | --- | --- |
| **Control** | **18±10 (96)** | **122** **± 29** | **60** **± 10** |
| **TNF-α 1ng/ml 12hr**  **+ 50% hypotonic** | **61±9 (80)*** | **348 ± 29##** | **120 ± 21** |
| **TNF-α 1ng/ml 12hr**  **+ 50% hypotonic**  **+ ruthenium red** | **6±4 (71) ***** | **15 ± 7###** | **70 ± 7** |
| **TNF-α 1ng/ml 16hr**  **+ 50% hypotonic** | **81±12 (111)**** | **565** **± 28##** | **118 ± 8** |
| **TNF-α 1ng/ml 16hr**  **+ 50% hypotonic**  **+ ruthenium red** | **12±4 (84) ***** | **41** **± 19###** | **61 ± 5** |

*** p<0.05, ** p<0.005, ## p<0.001 versus control; *** p<0.05, ### p<0.001 versus no ruthenium red**
